# A case report of familial catecholaminergic polymorphic ventricular tachycardia with a novel mutation in the ryanodine receptor 2

**DOI:** 10.1093/ehjcr/ytae652

**Published:** 2024-12-09

**Authors:** Yoshikuni Shoji, Satoshi Hayashida, Hikaru Masuda, Eizo Tachibana, Yasuo Okumura

**Affiliations:** Division of Cardiology, Kawaguchi Municipal Medical Center, 180 Nishiaraijyuku, Kawaguchi-shi, Saitama, 333-0833, Japan; Division of Cardiology, Kawaguchi Municipal Medical Center, 180 Nishiaraijyuku, Kawaguchi-shi, Saitama, 333-0833, Japan; Division of Cardiology, Kawaguchi Municipal Medical Center, 180 Nishiaraijyuku, Kawaguchi-shi, Saitama, 333-0833, Japan; Division of Cardiology, Kawaguchi Municipal Medical Center, 180 Nishiaraijyuku, Kawaguchi-shi, Saitama, 333-0833, Japan; Division of Cardiology, Department of Medicine, Nihon University School of Medicine, 30-1 Ohyaguchi-kamicho, Itabashi-ku, Tokyo, 173-8610, Japan

**Keywords:** Catecholaminergic polymorphic ventricular tachycardia, Ryanodine receptor (RYR2), Sudden cardiac death, Genetic testing, Case report

## Abstract

**Background:**

Catecholaminergic polymorphic ventricular tachycardia (CPVT) is suspected by clinical characteristics involving fatal arrhythmic events in childhood and adolescence. On the other hand, genetic testing is also important because the mutation site in the specific genes of CPVT is related to the risk of ventricular arrhythmias and gene penetrance.

**Case summary:**

We present a case of a 15-year-old male with a familial history of CPVT and a history of syncope at the age of 5. He experienced a cardiac arrest prompting out-of-hospital cardiopulmonary resuscitation, and his circulatory dynamics recovered. Multiple premature ventricular contractions inducted by a treadmill exercise test disappeared after a dosage of verapamil, flecainide, and nadolol, and a subcutaneous implantable cardioverter defibrillator was implanted. The novel pathogenic mutation with an insertion of histidine near the C-terminus of the RYR2 protein was identified by genetic testing in this case and his mother.

**Discussion:**

The RYR2 mutation in this case has not been previously reported and may be an intractable phenotype of CPVT associated with a strong familial history and fatal cardiac events even under adequate medical therapy.

**Learning points**

- Catecholaminergic polymorphic ventricular tachycardia (CPVT) is often seen as an isolated case without a known family history. Regardless of the presence or absence of a family history, CPVT is often driven by a single nucleotide polymorphism (SNP) mutation at a specific position in the RYR2 gene, which encodes a calcium-release channel on the cardiac sarcoplasmic reticulum.
- This case describes a heretofore unreported inheritable gene mutation associated with CPVT. Addition of histidine within the C-terminus of the cytoplasmic RYR2 protein was detected, and the variant may represent a medically intractable CPVT phenotype with potential for fatal cardiac events in patients receiving standard medical therapy.

## Introduction

Catecholaminergic polymorphic ventricular tachycardia (CPVT) is a rare, inheritable, potentially fatal arrhythmogenic disorder. The associated arrhythmic events are triggered by emotional stress or physical exertion and occur in patients with a normal electrocardiogram (ECG) and normal echocardiogram. The disorder is driven by genetic mutation, mainly to a mutation in either of two genes (RYR2 or CASQ2).^1^ The mutation results in release of Ca^2+^ from the sarcoplasmic reticulum (SR) of cardiac muscle due to a sudden catecholamine surge and thus causes ventricular arrhythmias, including ventricular fibrillation (VF), VT, premature ventricular contractions (PVCs), and even atrial arrhythmias. However, despite being a genetic disorder, CPVT often occurs as an isolated case where there is no family history.^2^ We encountered a teenager with CPVT for whom there was a family history, but the case was unique in that it was associated with a novel mutation in the RYR2 gene.

## Summary figure

**Figure:**
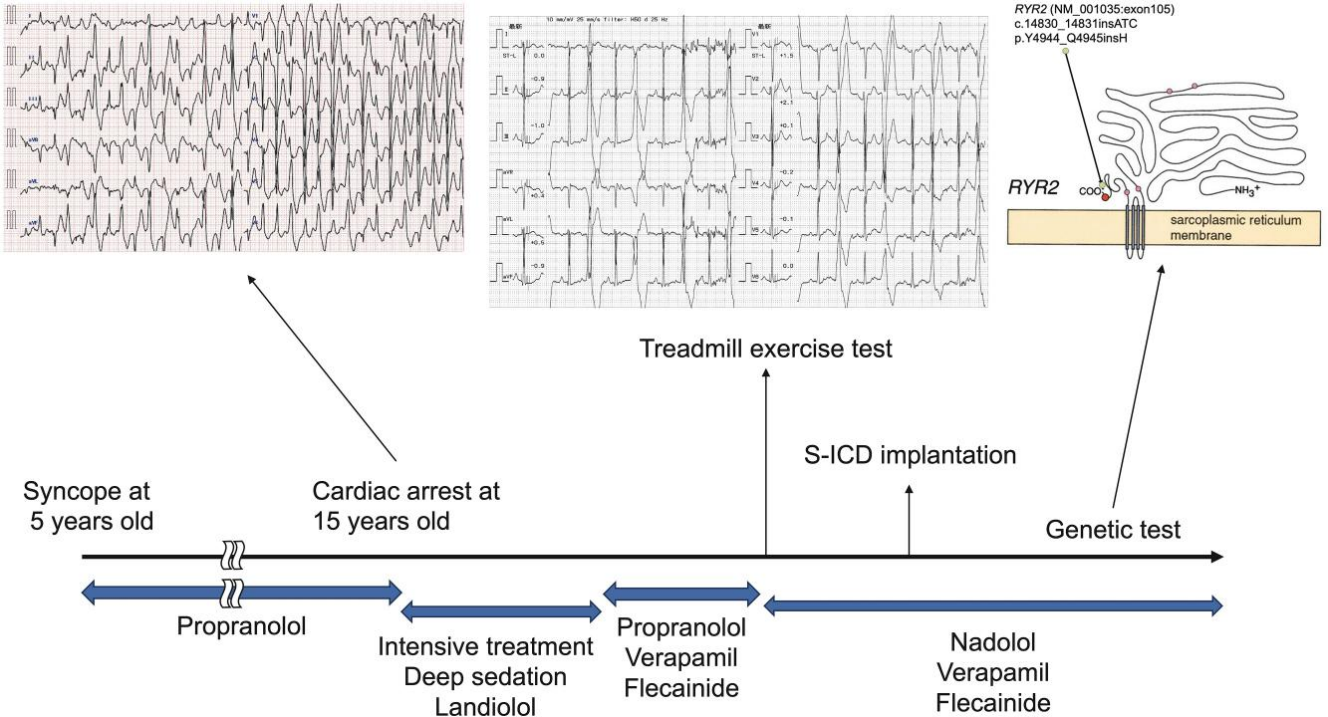


## Case presentation

**Figure 1:**
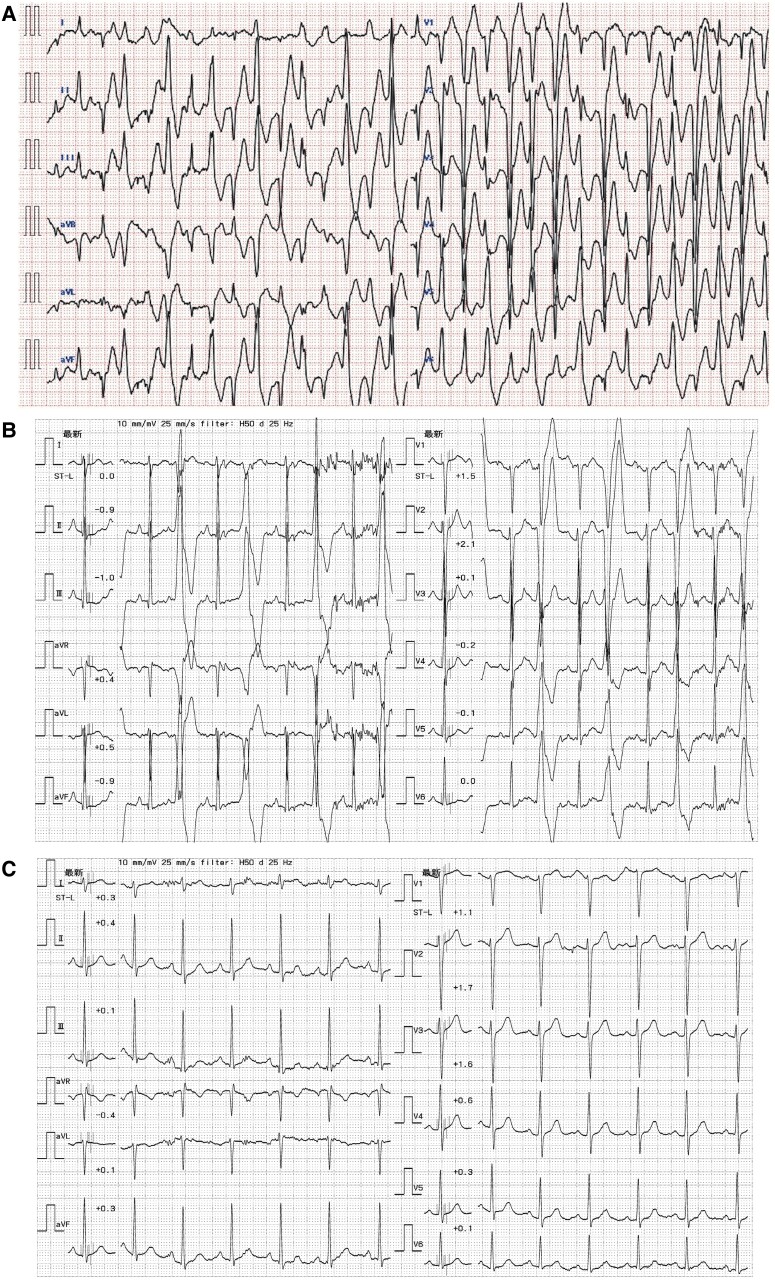
Twelve-lead electrocardiogram obtained (*A*) on arrival at our hospital after normalization of circulatory dynamics, (*B*) after rehabilitation during treadmill exercise test at 10.1 metabolic equivalents, and (*C*) after discharge during treadmill exercise test at 10.1 metabolic equivalents.

The case was that of a 15-year-old who, on his way to school, suffered cardiac arrest, prompting out-of-hospital cardiopulmonary resuscitation (CPR). He was admitted to our hospital, and a 12-lead surface ECG obtained on arrival (*Figure 1A*) showed multiple PVCs. Fortunately, his circulatory dynamics normalized. We learned that he had suffered an episode of syncope at age 5, had been diagnosed with CPVT at that time, and had been prescribed 80 mg/day of propranolol. We also learned that his mother, having suffered a cardiac arrest, had been diagnosed with CPVT. On arrival of the patient at our hospital, physical examination revealed a blood pressure of 131/88 mmHg, an irregular heart rate of 131 b.p.m., no heart murmur or pulmonary rales, no oedema, and no jugular vein distension. Laboratory test values were normal, with the exception of a high white blood cell count. Chest X-ray and echocardiography findings were normal; there was no cardiac dilation or structural heart disease, left ventricular function was normal, and there was no wall motion abnormality. The PVCs disappeared with continuous intravenous infusion of landiolol and deep sedation. Intensive therapy, including hypothermia and maintenance of circulatory dynamics, was undertaken, and the patient was eventually weaned from the ventilator and sedation. Because of the former diagnosis of CPVT, because no ST-T change or chest pain occurred under exercise ECG testing, and because cardiac magnetic resonance imaging ruled out old myocardial infarction and cardiomyopathy, we added verapamil and flecainide to the propranolol regimen to the maximum tolerable dose. After rehabilitation, a treadmill exercise test (TET) was performed, and left bundle branch block–type ventricular bigeminy was induced at an exercise workload of 10.1 metabolic equivalents (METs) (*Figure 1B*). Therefore, the β-blocker was changed from propranolol to nadolol, and a subcutaneous implantable cardioverter defibrillator (S-ICD) was implanted. The patient was discharged on Day 47.

**Figure 2:**
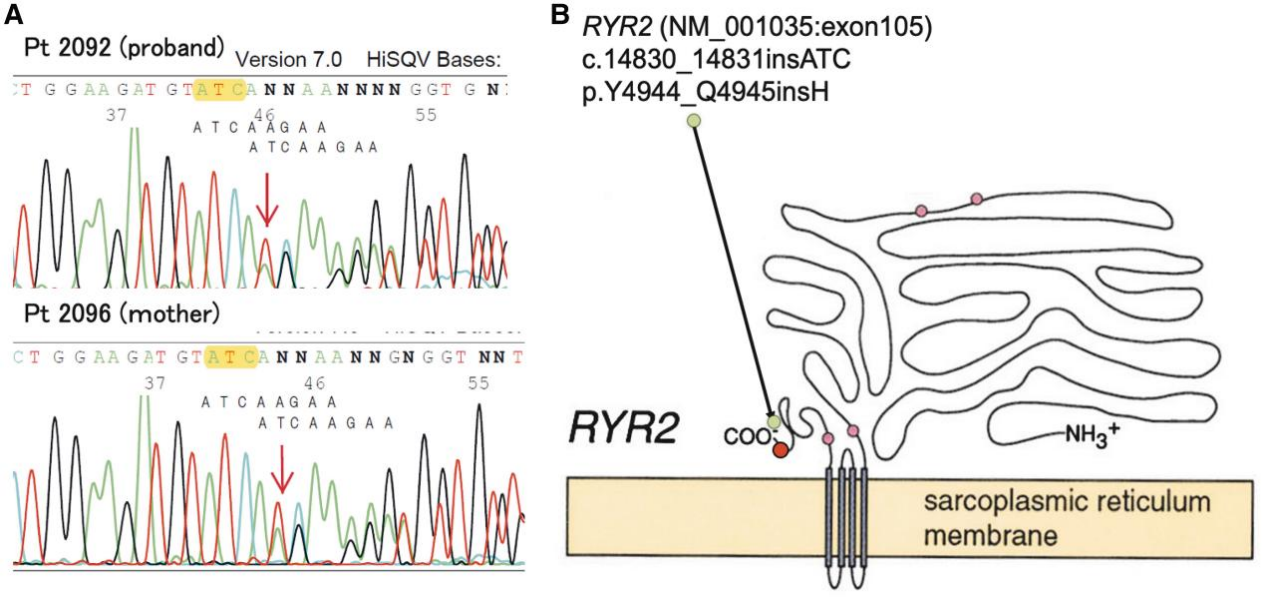
(*A*) Sequence analysis performed on our patient (proband) (upper left panel) and his mother (lower left panel) to identify any RYR2 mutation. (*B*) Schema of the RYR2 protein. The mutation in both individuals is located at NM_001035:exon105 near the C-terminus in the RYR2 protein.

On the basis of the patient’s history and the clinical course we observed, we attributed the cardiac event to CPVT. Genetic tests were performed to confirm CPVT in both the patient and his mother. A novel pathogenic insertion mutation c.14830_14831 ATC (NM_001035: exon105, RYR2 gene) was detected. The RYR2 gene encodes a ryanodine receptor (ryanodine receptor 2) found in cardiac muscle SR. This point mutation results in placement of histidine at position Y4944_Q4945 of the RYR2 protein (*Figure 2*) and has not been described in the gnomAD, 8.3KJPN, dbSNP, or ClinVar database. After the patient’s discharge, TET was again performed to measure his exercise capacity after addition of the anti-arrhythmic drugs, and no PVC or ventricular arrhythmia was induced at an exercise load of 10.1 METs (*Figure 1C*). Home monitoring was initiated for arrhythmia management, and non-sustained VT and paroxysmal atrial fibrillation were detected at times of emotional stress, but these terminated on their own without delivery of ICD shocks.

## Discussion

Catecholaminergic polymorphic ventricular tachycardia is a rare genetic condition that results in the occurrence of cardiac events such as syncope or sudden cardiac death during exercise or emotional stress in young patients with a normal echocardiogram and normal 12-lead ECG. Catecholaminergic polymorphic ventricular tachycardia is diagnosed, according to the criteria put forth in the HRS/EHRA/APHRS joint statement, in patients < 40 years of age with a structurally normal heart and normal ECG but found to have bidirectional VT, polymorphic ventricular premature beats, or VT induced by unexplained exercise or a catecholamine-related event, a pathogenic mutation, and/or a family history.^3^ There are two main types of CPVT, that attributable to mutation in the RYR2 gene and that attributable to mutation in the calsequestrin (CASQ2) gene, with these accounting for 50%–60% and 1% of CPVT cases, respectively.^1,4^ Cases of CPVT are often isolated cases; there is no family history even though the RYR2 mutation is an autosomal dominant mutation.^2^ The RYR2 protein is located on the SR membrane and controls intracellular Ca^2+^ release.^4^ Generally, mutation of the RYR2 gene in patients with CPVT is a single nucleotide polymorphism (SNP) at a specific position in the genome, and familial RYR2 variants are more likely to be located within the N-terminus domain than within the C-terminus domain.^5^ To our knowledge, this is the first report of a familial histidine insertion located near the C-terminus and within the cell cytoplasm. Because of the association between C-terminus RYR2 variants and an increased risk for ventricular arrhythmia,^6^ structural change near the C-terminus may explain the ease of inducibility and intractability in our patient’s case; histidine insertion in the RYR2 protein may induce increased release of store-overload-induced Ca^2+^ and/or a markedly reduced threshold for the occurrence of Ca^2+^ release.^5^ However, the exact mechanism is unknown because cases of CPVT involving histidine insertion in the RYR2 protein have not been reported previously.

The ICD implantation is an ESC Guidelines Class I recommendation as a secondary intervention for prevention of fatal arrhythmic events that can occur in CPVT patients after aborted cardiac arrest despite application of β-blocker therapy.^1^ However, VF storm or inappropriate ICD shocks are often seen in CPVT cases because of excessive catecholamine release. Since the first comprehensive CPVT series was described in 1995,^7^ β-blockers administered at maximum dosage have been considered the most effective therapy for preventing ventricular arrhythmia or sudden cardiac death. Underdosing of β-blockers has been often noted in the patients with CPVT,^8^ but in the case we report, propranolol had been administered at 80 mg/day before the cardiac event, despite the patient’s small stature, whereas the mean dose given to 21 patients surveyed was 52–64 mg/day in the previous report.^9^ Therefore, for our patient, we added verapamil and flecainide after the hospitalization period because it has been reported that the addition of these drugs to β-blocker can prevent cardiac events better than β-blocker alone.^10,11^ In addition, the β-blocker propranolol was changed to nadolol because it has been reported that nadolol is most effective in preventing cardiac events.^12^ To assess efficacy of this drug combination, TET was performed after the patient’s discharge, and there was no PVC at an exercise workload of 10.1 METs. The ESC Guidelines recommend left cardiac sympathetic denervation (LCSD) for patients with CPVT in whom pharmacotherapy is not effective,^1^ but we decided against LCSD because of the effectiveness of the additional pharmacotherapy in our patient. Many patients with CPVT are young, like ours, and we chose to implant an S-ICD considering the patient’s ongoing growth, the ineffectiveness of anti-tachycardia pacing in cases of CPVT, and the risk of lead-related complications associated with long-term use of a transvenous ICD.

## Conclusion

Importance of the CPVT case reported herein lies in the detection of a heretofore unreported gene mutation associated with the disorder. This inheritable CPVT variant may represent a novel and medically intractable CPVT phenotype, with potential for fatal cardiac events even in patients receiving standard medical therapy.

## Data Availability

The data underlying this article are available in the article.

## Lead author biography

Dr Yoshikuni Shoji received his MD degree from Saitama Medical University in 2017. Since 2019, he has been working in the Division of Cardiology, Kawaguchi Municipal Medical Center, as a cardiologist. His areas of most interest are coronary intervention and echocardiography.

## Notes

**Consent:** The authors confirm that written consent for submission and publication of this case report, including the images and associated text, has been obtained from the patient in line with the COPE guidelines.

**Funding:** None declared.
